# Twenty Whole-Genome *Bacillus* sp. Assemblies

**DOI:** 10.1128/genomeA.00958-14

**Published:** 2014-10-09

**Authors:** H. E. Daligault, K. W. Davenport, T. D. Minogue, K. A. Bishop-Lilly, S. M. Broomall, D. C. Bruce, P. S. Chain, S. R. Coyne, K. G. Frey, H. S. Gibbons, J. Jaissle, G. I. Koroleva, J. T. Ladner, C.-C. Lo, C. Munk, G. F. Palacios, C. L. Redden, C. N. Rosenzweig, M. B. Scholz, S. L. Johnson

**Affiliations:** a Los Alamos National Laboratory (LANL), Los Alamos, New Mexico, USA; b Diagnostic Systems Division, U.S. Army Medical Research Institute of Infectious Diseases (USAMRIID), Fort Detrick, Maryland, USA; c Naval Medical Research Center (NMRC)-Frederick, Fort Detrick, Maryland, USA; d Henry M. Jackson Foundation, Bethesda, Maryland, USA; e U.S. Army Edgewood Chemical Biological Center (ECBC), Aberdeen Proving Ground, Aberdeen, Maryland, USA; f Center for Genome Sciences (CGS), USAMRIID, Fort Detrick, Maryland, USA

## Abstract

Bacilli are genetically and physiologically diverse, ranging from innocuous to highly pathogenic. Here, we present annotated genome assemblies for 20 strains belonging to *Bacillus anthracis*, *B. atrophaeus*, *B. cereus*, *B. licheniformis*, *B. macerans*, *B. megaterium*, *B. mycoides*, and *B. subtilis*.

## GENOME ANNOUNCEMENT

The genus *Bacillus* comprises Gram-positive rod-shaped endospore-forming bacteria that are either facultative or obligate aerobes. Members are both phylogenetically and physiologically diverse and are found in a vast array of environmental, symbiotic, and pathogenic roles and habitats, including the category A pathogen *Bacillus anthracis* (1, 2). Here, we present the genome sequences of 20 *Bacillus* isolates (various species), either in completed or scaffolded status.

High-quality genomic DNA was extracted from purified isolates of each strain using QIAgen Genome Tip-500 at the U.S. Army Medical Research Institute of Infectious Diseases, Diagnostic Systems Division (USARMIID-DSD). Specifically, 100-mL bacterial cultures were grown to stationary phase and nucleic acid was extracted per the manufacturer’s recommendations, with one minor variation. For BSL3 *Bacillus anthracis*, all cultures were lysed overnight to ensure sterility of the resulting extracted material. If sterility was not achieved, the nucleic acid was passed through a 0.45-µm filter and rechecked for viable organisms before removal from the BSL3 suite. Sequence data for each draft genome were generated using a combination of Illumina and 454 technologies (3, 4). For each genome, we constructed and sequenced an Illumina library of 100-bp reads at high coverage (ranging from 136 to 950) and a separate long-insert paired-end (insert size ranging from 7.19 to 10.6 kb) library (Roche 454 Titanium or Illumina platform). The two data sets were assembled together in Newbler (Roche), and the consensus sequences were computationally shredded into 2-kbp overlapping fake reads (shreds). The raw reads were also assembled in Velvet and those consensus sequences computationally shredded into 1.5-kbp overlapping shreds (5). Draft data from all platforms were then assembled together with Allpaths, and the consensus sequences computationally shredded into 10-kbp overlapping shreds (6). We then integrated the Newbler consensus shreds, Velvet consensus shreds, Allpaths consensus shreds, and a subset of the long-insert read pairs using parallel Phrap (High Performance Software, LLC). Possible misassemblies were corrected, and some gap closure was accomplished with manual editing in Consed (7–9).

Automatic annotation for each genome utilized an Ergatis-based workflow at LANL with minor manual curation. Each genome is available in NCBI (accession numbers listed in Table 1), and raw data can be provided upon request. In-depth comparative analyses of these and other genomes are currently under way and will be published in subsequent reports.

**TABLE 1:** Strain-identifying information and basic statistics on assemblies and annotations

Strain	Accession no. (no. of contigs)^*a*^	Genome size (bp)	%GC	Draft coverage	No. of CDSs^*b*^	No. of tRNAs	No. of rRNAs
*Bacillus anthracis*							
2000031021	CP007618 Chr. CP007617 pXO2	5,331,737	35.2	303	5,509	96	33
BA0052	CP007704 Chr. CP007703 pXO1 CP007702 pXO2	5,504,355	53.3	897	5,777	95	33
Delta Sterne	CP008752 Chr	5,226,650	35.4	579	5,479	107	32
Pasteur-like	JNOD00000000 WGS (18)	5,285,189	35.3	305	5,564	75	18
Scotland A.Br.003	JMPV00000000 WGS (8)	5,487,216	35.2	305	5,757	82	22
Vollum	CP007666 Chr CP007665 pXO1 CP007664 pXO2	5,506,189	35.4	315	5,774	94	33
Zimbabwe 89	JMPU00000000 WGS (17)	5,459,523	35.1	437	5,795	79	14
*Bacillus atrophaeus*							
var. *globigii*	CP007640 Chr	4,174,560	43.1	402	4,098	80	24
*Bacillus cereus*							
4342	JMPY00000000 WGS (19)	5,260,613	35.4	445	5,319	122	24
10876	JMPW00000000 WGS (26)	5,993,683	34.8	557	6,013	100	20
13061	JMPX00000000 WGS (53)	5,465,916	35.3	319	5,626	113	18
F1-15	JMSG00000000 WGS (40)	5,596,712	35.3	975	5,754	110	20
*Bacillus licheniformis*							
12759	JMPZ00000000 WGS (18)	4,387,510	45.8	556	4,348	80	13
*Bacillus megaterium*							
de Bary 1884	JMQB00000000 WGS (31)	5,618,359	37.7	269	5,756	114	19
*Bacillus mycoides*							
219298, BA0098	CP007621 Chr CP007622 pBHG01 CP007623 pBHG02 CP007624 pBHG03 CP007625 pBHG04 CP007626 pBHG05	5,675,302	35.6	314	5,678	115	42
BHP	JMQC00000000 WGS (12)	5,875,917	35.3	318	5,966	107	42
Flugge 10206	JMQD00000000 WGS (121)	5,374,126	35.4	398	5,580	40	5
*Bacillus subtilis*							
NRS231	JMNA00000000 WGS (4)	4,042,815	44.0	180	3,987	90	30
var. Niger PCI246	JMTJ00000000 WGS (7)	4,158,658	43.2	329	4,105	87	17
*Paenibacillus macerans*							
8244	JMQA00000000 WGS (64)	7,331,450	53.0	149	6,561	75	10

a Chr, chromosome.

b CDSs, coding sequences.

### Nucleotide sequence accession numbers.

Genome accession numbers to public databases are listed in Table 1.
